# Ewing’s sarcoma of the mediastinal extraosseous site: a case report of a rare occurrence with literature review

**DOI:** 10.3389/fonc.2025.1578456

**Published:** 2025-06-18

**Authors:** Hong Zhou, Meng Su, Han-Fang Wang, Si-Qi Feng, Wen-Qian Jiang, Xiao-Rong Li

**Affiliations:** Radiology Department, General Hospital of Southern Theater Command, People‘s Liberation Army (PLA), Guangzhou, China

**Keywords:** extraskeletal Ewing’s sarcoma(EES), mediastinum, case report, rare site, computed tomography

## Abstract

**Objective:**

To present a rare case of mediastinal extraosseous Ewing sarcoma(EES), broadening the body of knowledge already in existence on EES. This paper intends to give doctors more diagnostic and therapeutic understanding

**Methods:**

The patient’s imaging data was analyzed retrospectively. We identified and compiled the distinctive imaging characteristics of mediastinal EES by combining these results with histopathological evaluation and a review of relevant literature.

**Key findings:**

Typical imaging features of mediastinal EES include a large, bulky mass with irregular shape and lobulated, poorly defined margins. On computed tomography (CT), it often shows heterogeneous density along with infiltration of nearby organs and anatomical structures.

**Conclusion:**

Mediastinal EES is a rare and clinically challenging diagnosis. However, recognition of its characteristic imaging features may facilitate radiological diagnosis. Nevertheless, histopathological confirmation remains the gold standard for definitive diagnosis.

## 1 Introduction

Ewing’s sarcoma ranks as the second most prevalent primary malignant bone tumor in children and adolescents. Although it predominantly arises within the skeletal system, this aggressive neoplasm can also manifest in extraosseous locations, which is called extraosseous Ewing’s sarcoma (EES) (1). This case represents a primary extraosseous Ewing’s sarcoma (EES) arising within the mediastinum. EES is distinguished by its heterogeneous distribution of primary sites, pronounced invasiveness, and a propensity for local recurrence and distant metastasis, which collectively confer a dismal prognostic outlook. These characteristics make it difficult to diagnose EES early and accurately, which frequently results in a high rate of diagnostic errors.

A rare instance of mediastinal EES is described in this paper. We hope to clarify important diagnostic imaging characteristics, treatment approaches, and prognostic indicators unique to EES by combining this case with an extensive analysis of the body of current literature. Our goal is to improve patient management and outcomes for those suffering from this uncommon and aggressive cancer by providing physicians with better knowledge about the preoperative diagnostic assessment, treatment selection, and efficacy monitoring of EES.

## 2 Case presentation

Presenting with a 2-week history of nocturnal dry cough accompanied by pharyngeal irritation unresponsive to outpatient anti-inflammatory treatment, was a 33-year-old male. Symptoms had progressed significantly and included coughing, dyspnea, and chest tightness that led him to tertiary center hospitalization. Clinical degradation continued despite symptomatic treatment (anti-inflammatories, expectorants, bronchodilators), thus, referral to our institution’s cardiothoracic surgery department was needed after 2 days.

When admitted, physical examination was normal. Laboratory studies turned up the following unusual values: With albumin within normal limits (39.6 g/L; reference: 35–55 g/L), serum total protein was rather lowered at 62 g/L (reference range). Tumor markers (CEA, AFP) revealed no appreciable anomalies.

**Figure 1:**
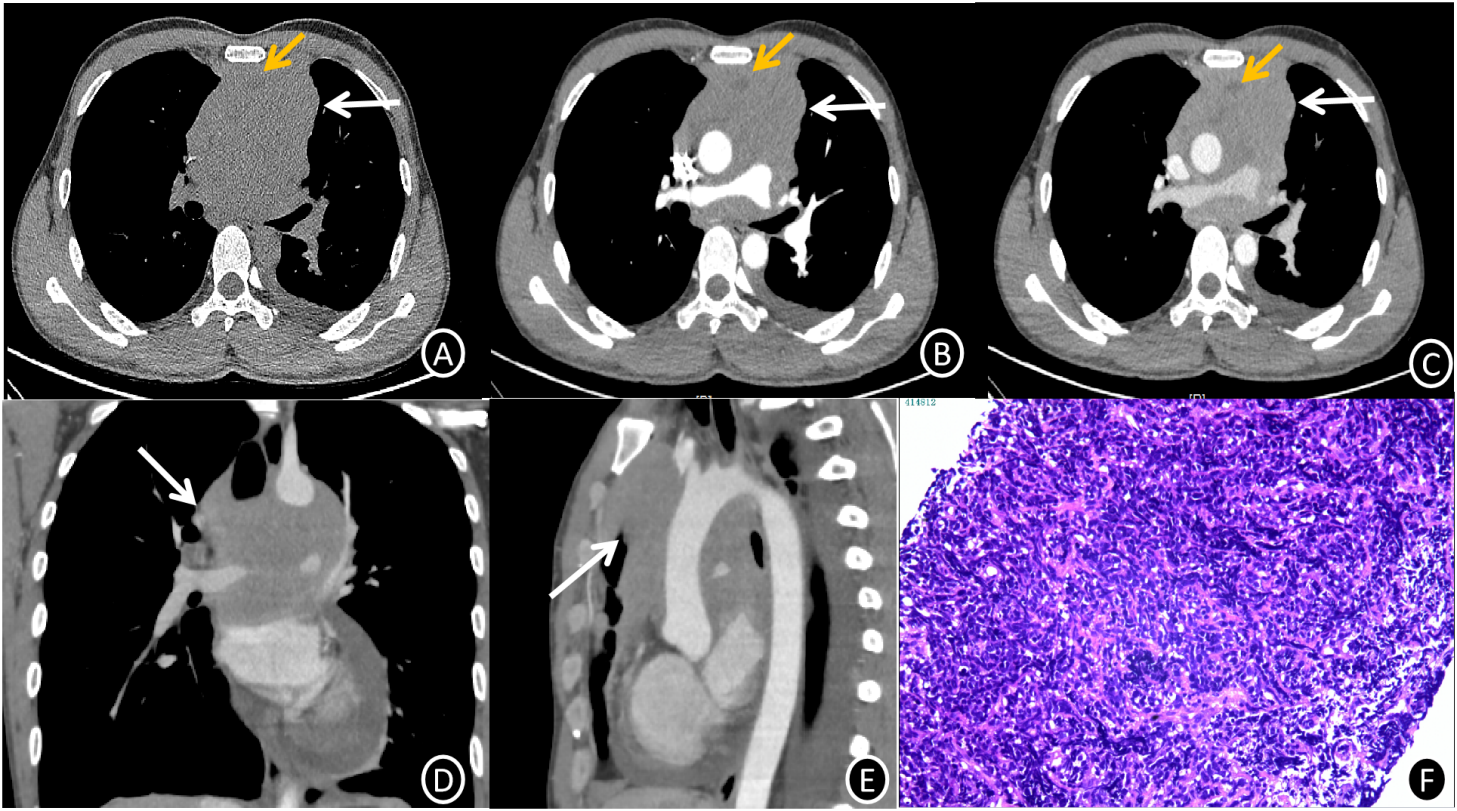
**(A-C)** Mediastinal EES CT images (1.0 mm slice thickness) showing axial non-contrast **(A)**, arterial phase **(B)**, and venous phase **(C)**. A lobulated, mostly iso-dense soft tissue mass (white arrow) with internal heterogeneous hypodense foci and vascular encasement can be seen in the anterior-superior mediastinum on non-contrast imaging **(A)**. Progressive heterogeneous enhancement is noted on post-contrast series **(B, C)**, with mean attenuation values of 47 HU (non-contrast), 57 HU (arterial phase), and 72 HU (venous phase). Non-enhancing necrotic regions are highlighted (yellow arrows). **(C-E)** Venous phase axial **(C)**, coronal **(D)**, and sagittal **(E)** reformations (1.0 mm slice thickness) illustrate circumferential vascular involvement (white arrows) with loss of fat planes between the mass, great vessels, and pericardium. **(F)** Photomicrograph of mediastinal EES (H&E stain, ×200 magnification) reveals infiltrative nests and cords of small hyperchromatic cells with high nuclear-to-cytoplasmic ratios, brisk mitotic activity, and focal necrosis within a fibrotic stroma.

**Figures 1A–E**; Contrast-Enhanced CT: The anterior-middle mediastinum was occupied by a 10 cm × 9.7 cm × 9.6 cm heterogeneously enhancing soft tissue mass with lobulated margins, peripheral fat stranding, and vascular encasement. With accompanying involvement of the ascending aorta, aortic arch, bilateral pulmonary arteries, and right inferior pulmonary vein, the lesion showed patchy non-enhancing necrotic areas and internal vasculature. The mean CT attenuation values across the non-contrast and contrast-enhanced phases were approximately 47, 57, and 72 Hounsfield units, respectively. Along with loss of the pericardial fat plane, noted were compression of the superior vena cava (SVC), left main bronchus, and right pulmonary artery.

**Figure 1F** 6 days after admission, histologic analysis of an ultrasound-guided core needle biopsy, showed nests and cords of small, hyperchromatic cells with high nuclear-to-cytoplasmic ratios and rapid mitotic activity, which are indicative of malignant neoplasm. Diffuse membranous CD99(+), focal FLI-1(+), and scattered TdT(+) nuclear staining were shown by immunohistochemistry, while epithelial (CK), lymphoid (CD20, CD79a, CD3, CD5), neuroendocrine (CgA, Syn), and myogenic (desmin) markers showed negative staining. EBER *in situ* hybridization was negative, but the Ki67 proliferation index was close to 90%. These results validated the extraosseous Ewing sarcoma (EES) diagnosis.

**Figure 2:**
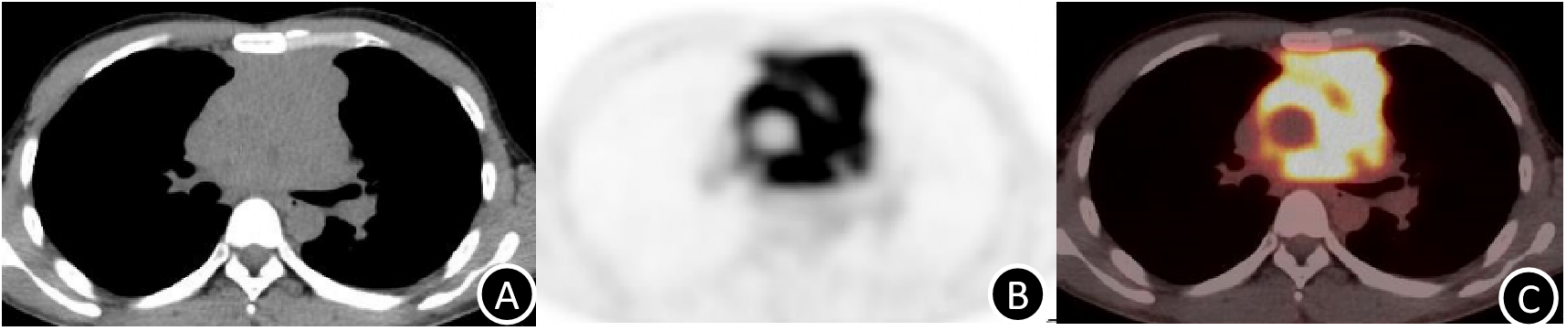
**(A-C)** PET/CT imaging (4.0 mm slice thickness) demonstrates intense FDG avidity (SUVmax 11.6) within the heterogeneously enhancing mediastinal mass, corresponding to regions of viable tumor (arrow). .

PET/CT; **Figures 2A–C** Involving the anterior-middle mediastinum, encasing major vascular structures (aortic arch, bilateral pulmonary arteries, right inferior pulmonary vein), FDGavidmetabolistically active lesions (SUVmax 11.6) abutting the SVC and pericardium. Radiologic differential diagnosis favored lymphoma; biopsy was advised for confirmation.

**Figure 3:**
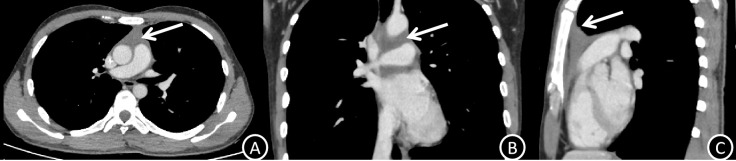
**(A–C)** Post-treatment venous phase CT images (axial, coronal, and sagittal planes) demonstrate marked regression of the anterior mediastinal mass (white arrows) compared with pre-treatment imaging, consistent with partial response to systemic therapy.

The patient started systemic chemotherapy after informed consent and a multidisciplinary discussion. The effectiveness of the treatment was confirmed by post-induction imaging (**Figures 3A–C**), which showed a partial response with tumor regression and symptomatic improvement.

## 3 Discussion

Ewing sarcoma of bone (ESB), extraosseous Ewing sarcoma (EES), primitive neuroectodermal tumor of soft tissue (PNET), and Askin tumor (malignant small cell tumor of the thoracopulmonary region) are among the various entities that are included in the Ewing sarcoma family of tumors (ESFT). The small round cell morphology of these tumors is one of their common characteristics (2). The most frequent primary sites for EES, according to Zhang et al., are the paravertebral regions, lower extremities, and chest wall; the upper extremities, hip joints, and pelvic cavity are less frequently affected (3). Although anterior mediastinal EES has been reported in a few case reports (e.g., Caltavituro et al., Cui et al.), mediastinal primary sites are extremely uncommon (1, 4).

Although about 30% of cases occur in adults (over 20 years old), EES primarily affects adolescents, peaking between the ages of 10 and 15 (5). Clinically, these tumors have a poor prognosis and aggressive growth. They frequently present with vague initial symptoms (like soft tissue pain or swelling) that cause the diagnosis to be delayed until the tumor is in an advanced stage. Early in the course of the disease, metastatic spread is common, especially to the lungs and bones, and relapse rates are high even after treatment (6). Histopathologically, EES shows thick layers of tiny, round cells that are nested and cord-like, sometimes forming Homer-Wright rosettes. Neuroendocrine differentiation markers, such as NSE, S-100, neurofilament, synaptophysin, and chromogranin A, are commonly found in immunohistochemical profiles; additionally, positive expression of CD99 and vimentin is considered diagnostically significant for confirming the diagnosis (7).

Multifocal large-to-giant solid masses are the usual presentation of extraosseous Ewing sarcoma (EES). EES exhibits nonspecific imaging characteristics on radiography: computed tomography (CT) shows it as isodense or slightly hypodense lesions, and magnetic resonance imaging (MRI) shows hyperintensity on T2-weighted imaging (T2WI) and isointense to mildly hypointense signals on T1-weighted imaging (T1WI). Heterogeneous density/signal intensity may arise from associated hemorrhage, necrosis, cystic/mucoid degeneration, particularly in larger tumors; smaller lesions often exhibit homogeneous characteristics. Post-contrast administration, most lesions demonstrate marked to moderate enhancement (8). These atypical imaging profiles frequently complicate differentiation from other soft tissue neoplasms.

However, EES has diagnostic characteristics such as aggressive invasion of adjacent organs or structures, irregular, lobulated masses with unclear margins, and inherent heterogeneity in larger tumors (8). Despite these unique characteristics, diagnostic difficulties remained in this instance because the interpreting team’s initial radiologic interpretation was unable to identify the constellation of classic imaging features, which may have been caused by their lack of familiarity with EES.Thymoma, germ-cell tumors (GCTs), and lymphoma are all included in the differential diagnosis for anterior mediastinal lesions; each has unique radiologic and clinical characteristics. Thymoma, the most prevalent primary anterior mediastinal tumor in adults (50–80% of cases), usually appears as a solid mass in the anterior-superior mediastinum that is round and well-circumscribed. Thymomas, which are most commonly diagnosed in middle-aged adults, can coexist with paraneoplastic syndromes like myasthenia gravis. On radiography, these tumors show preserved peritumoral fat planes, homogeneous density, and mild post-contrast enhancement. Invasive features, such as lobulated margins, heterogeneous density, or calcifications, indicate malignant transformation.

Another important distinction is germ-cell tumors, especially teratomas. Teratomas, which are mostly found in the middle mediastinum, are complex cystic-solid masses with mixed densities caused by calcifications, fat, and dermal appendages (9). Although malignant degeneration may cause irregularity or heterogeneous enhancement, smooth margins and mild enhancement of solid components are typical.Lymphoma, while more commonly involving the middle mediastinum, may arise anteriorly and typically affects middle-aged adults, with a female predilection (10). Clinical manifestations may include B symptoms (e.g., fever, night sweats). Imaging reveals irregular contours, homogeneous density, and vascular encasement on post-contrast studies, though lymphadenopathy often dominates the radiologic picture. Accurate diagnosis is still possible through careful examination of distinguishing imaging characteristics, even though it can be difficult to distinguish mediastinal EES from common anterior mediastinal tumors.

Systemic therapy (chemotherapy and radiation) is the main treatment modality for EES, while multimodal therapy combines surgery, radiotherapy, and chemotherapy. Neoadjuvant chemotherapy increases resectability by eliminating micrometastases and lowering the primary tumor burden. Only in cases of incurable disease, insufficient surgical margins, or a poor response to systemic therapy is radiotherapy used.

## 4 Conclusion

In conclusion, extraosseous Ewing sarcoma (EES) is a small round cell neoplasm that is uncommon, extremely aggressive, and preferentially found in extraosseous locations. Due to mild early symptoms, mediastinal involvement is extremely rare, and clinical presentation is usually nonspecific and postponed until advanced stages. On radiography, EES usually appears as a large, irregular soft tissue mass with a heterogeneous density and signal intensity. Cystic or necrotic components and aggressive invasion of adjacent organs are often present. Lobulated margins, poorly defined interfaces, and inherent heterogeneity in larger lesions are characteristics of imaging. Preoperative diagnosis, staging, treatment planning, and evaluation of therapeutic response all heavily rely on imaging. For a conclusive diagnosis in cases with unclear imaging results, histopathologic correlation is still required.

## Data Availability

The original contributions presented in the study are included in the article/**Supplementary Material**. Further inquiries can be directed to the corresponding author.

## Supplementary material

Supplementary Video 1 Three-dimensional volume-rendered reconstruction illustrates the irregular, green-pseudocolor-encoded tumor (anterior aspect) with indistinct margins relative to adjacent great vessels, highlighting its locally aggressive behavior.

## References

[1] Caltavituro A Buonaiuto R Salomone F Morra R Pietroluongo E De Placido P . Extraskeletal Ewing’s sarcoma of the mediastinum: Case report. Front Oncol. (2023) 13:1074378. doi: 10.3389/fonc.2023.1074378 36776337 PMC9911166

[2] Tarek N Said R Andersen CR Suki TS Foglesong J Herzog CE . Primary Ewing sarcoma/primitive neuroectodermal tumor of the kidney: the MD anderson cancer center experience. Cancers (Basel). (2020) 12:2927. doi: 10.3390/cancers12102927 33050651 PMC7599660

[3] Zhang GH Lin JM He ZY Yuan XJ Li G Gan XR . A case of giant Ewing’s sarcoma (EES)/primitive neuroectodermal tumor (PNET) of the cervicothoracic junction in children with incomplete paralysis of both lower limbs: Case report and literature review. Front Surg. (2022) 9:1066304. doi: 10.3389/fsurg.2022.1066304 36684168 PMC9852823

[4] Cui M Zhai D Liu Y Zhou X Wang T Wang L . Case report: Primary mediastinal Ewing’s sarcoma presenting with chest tightness. Front Oncol. (2022) 12:1020339. doi: 10.3389/fonc.2022.1020339 36815073 PMC9940310

[5] Durer S Gasalberti DP Shaikh H . Ewing sarcoma. (2025). 32644609

[6] Applebaum MA Goldsby R Neuhaus J Dubois SG . Clinical features and outcomes in patients with secondary Ewing sarcoma. Pediatr Blood Cancer. (2013) 60(4):611–5. doi: 10.1002/pbc.24251 PMC3488141 22847990

[7] Liu J Zhao YL Song SQ Li ZH Li PL . Primitive neuroectodermal tumors: a clinical and radiological analysis of six cases. Quant Imaging Med Surg. (2019) 9:722–9. doi: 10.21037/qims.2019.03.16 PMC6511716 31143663

[8] Wright A Desai M Bolan CW . Extraskeletal Ewing sarcoma from head to toe: multimodality imaging review. Radiographics. (2022) 42:1145–60. doi: 10.1148/rg.210226 35622491

[9] Carter BW Benveniste MF Madan R Godoy MC De Groot PM Truong MT . ITMIG classification of mediastinal compartments and multidisciplinary approach to mediastinal masses. Radiographics. (2017) 37:413–36. doi: 10.1148/rg.2017160095 28129068

[10] Savage KJ . Primary mediastinal large B-cell lymphoma. Blood. (2022) 140:955–70. doi: 10.1182/blood.2020008376 34496020

